# Intraventricular Hemorrhage in Premature Infants with Respiratory Distress Syndrome Treated with Surfactant: Incidence and Risk Factors in The Prospective Cohort Study

**DOI:** 10.34763/devperiodmed.20172104.328335

**Published:** 2018-01-02

**Authors:** Ewa Helwich, Magdalena Rutkowska, Renata Bokiniec, Ewa Gulczyńska, Roman Hożejowski

**Affiliations:** 1 Klinika Neonatologii, Instytutu Matki i Dziecka, Warszawa, Polska; 2 Klinika Neonatologii, Warszawski Uniwersytet Medyczny, Warszawa Polska; 3 Klinika Neonatologii, IP – Centrum Zdrowia Matki Polki w Łodzi, Łodzi Polska; 4 Dział Medyczny, Chiesi Poland, Warszawa, Polska

**Keywords:** intraventricular hemorrhage (IVH), antenatal corticosteroids, respiratory distress syndrome (RDS), preterm newborns, krwawienia dokomorowe (IVH), glukokortykoidy prenatalne, zespół zaburzeń oddychania (RDS), noworodki urodzone przedwcześnie

## Abstract

**Background:**

Intraventricular hemorrhage (IVH) is a common pathology in preterm infants with extremely and very low birth weight. It is particularly often seen in newborns with Respiratory Distress Syndrome (RDS).

**Aim:**

To assess the incidence of IVH in preterm newborns with RDS treated with surfactant, and to identify factors that might reduce the risk of IVH in this population.

**Material and methods:**

This multicenter, prospective cohort study is part of the “Neo-pro” study project. The investigations were carried out in 936 newborns, including 652 survivors. We enrolled a consecutive sample of infants born before 32 weeks’ gestation. IVH was diagnosed with trans-fontanel ultrasonography, performed according to the approved standards and classified according to Papile’s grading system.

**Results:**

Intraventricular hemorrhage was diagnosed in 462/936 infants (49.4%), and in 43.3% of the survivors. Grade 3 and 4 IVH occurred in 14.8% and 13.8% of the infants, respectively, and in 10.6% and 5.7% of the survivors. Lack of antenatal application in mothers of corticosteroids increased the incidence rate of severe IVH from 14.2% to 22.1% (p=0.0087). The risk of IVH was reduced with early (from the first day of life) initiation of caffeine citrate (OR: 0.63, 95% CI: 0.45-0.88), delivery by cesarean section (OR: 0.50, 95% CI: 0.36-0.69), and the risk of severe IVH - from treatment with antenatal corticosteroids (OR: 0.58, 95% CI: 0.39-0.87). The most significant factor which increased the risk of hemorrhage was invasive mechanical ventilation (OR: 2.90, 95% CI: 2.07-4.07). The risk was further increased if the duration of mechanical ventilation was greater than seven days (OR: 3.02, 95% CI: 2.21-4.12).

**Conclusions:**

The incidence of IVH in newborns with RDS is significant and the risk of IVH is increased by mechanical ventilation. Antenatal exposure to corticosteroids and delivery by cesarean section have a protective effect, and the former also reduces the risk of the most severe manifestations of IVH. Caffeine citrate initiated from the first day of life is another protective strategy.

## Streszczenie

Krwawienia około-dokomorowe są częstą patologią występująca u noworodków urodzonych przedwcześnie z ELBW i VLBW. Szczególnie często są stwierdzane u noworodków chorych z zespołem zaburzeń oddychania.

**Celem:**

badania było stwierdzenie częstości występowania krwawień około-dokomorowych u noworodków z zespołem zaburzeń oddychania leczonych surfaktantem oraz identyfikacja czynników obniżających ryzyko krwawień wewnątrzczaszkowych w tej populacji.

**Materiał i metody:**

Wieloośrodkowe, prospektywne, kohortowe badanie było realizowane w ramach projektu NeoPro. Badanie wykonano u 936 noworodków urodzonych przed ukończeniem 32 tygodni ciąży.

Krwawienia dokomorowe i okołokomorowe diagnozowano w oparciu o ultrasonograficzne badania przezciemiączkowe wykonywanymi zgodnie z obowiązującymi standardami i klasyfikowano w czterech stopniach ciężkości według Papile’a.

**Wyniki:**

Krwawienia stwierdzono u 462/936 dzieci (49,4%) i 43,3% dzieci które przeżyły. Krwawienie III i IV stopnia wystąpiły odpowiednio u 14,8% i 13,8% noworodków w całej grupie oraz 10,6% i 5,7% noworodków które przeżyły. Niezastosowanie u matek prenatalnie kortykosteroidów zwiększyło częstość rozległych krwawień z 14,2% do 22,1% (p=0,0087). Ryzyko krwawień dokomorowych opóźniało wczesne (od pierwszej doby) rozpoczęcie terapii kofeiną (OR 0,63, 95% CI: 0,45-0,88), poród przez cięcie cesarskie (OR 0,50, 95% CI: 0,36-0,69), a ciężkich krwawień – steroidoterapia prenatalna (OR 0,58, 95% CI: 0,39-0,87). Czynnikami zwiększającymi ryzyko krwawień było stosowanie wentylacji inwazyjnej (OR 2,90, 95% CI: 2,07-4,07), w szczególności trwającej ponad 7 dni (OR 3,02, 95% CI: 2,21-4,12).

**Wnioski:**

Częstość krwawień wewnątrzczaszkowych (IVH) u noworodków z zespołem zaburzeń oddychania jest znaczna, a ich ryzyko zwiększa wentylacja mechaniczna. Do czynników zmniejszających ryzyko IVH, w tym ciężkiego stopnia, w badanej populacji należy indukcja farmakologiczna płodu glukokortykoidami podawanymi matce w sytuacji zagrożenia porodem przedwczesnym. Wczesne (od pierwszej doby życia) rozpoczęcie leczenia kofeiną zmniejszyło ryzyko krwawień do oun.

## Introduction

Intraventricular hemorrhage (IVH) is the most frequent form of early brain injury in preterm newborns. A significant clinical and prognostic importance can be attributed to both extensive grade 3 hemorrhage, in which blood fills more than 50% of the lumen of the lateral ventricle causing it to enlarge, and periventricular hemorrhagic infarction (PVHI), also known as grade 4 IVH [1].

The main cause of the occurrence of IVH is immaturity and lack of autoregulation of cerebral vessels, observed in infants born before 32 gestational weeks. In this population, the highest risk applies to infants with respiratory and circulatory failure in the course of neonatal Respiratory Distress Syndrome (RDS). Hypotension, disturbance in the cerebral blood flow, and secondary reperfusion are the causative factors [2, 3].

Since most bleedings occur in the first three days of life, it is important to identify factors that are already working in the prenatal and early neonatal periods, which could reduce the risk of this complication [4]. This is particularly important in the prevention of extensive bleedings (grade 3/PVHI), because they increase the risk of abnormal neurological development, such as cerebral palsy, cognitive, and behavioral abnormalities.

## Aim

To determine the incidence of IVH in neonates with RDS treated with surfactant, and the identification of risk factors for IVH in this population.

## Material and methods

The study was prospective and carried out within the framework of a wider project, the NeoPro study.

The investigation was carried out in 936 premature newborns, with 652 survivors. The study population consisted of 55.3% males, while regarding the place of delivery − the majority of the infants were inborn (88.9%). The median gestational age was 28 weeks (IQR: 26.3-30.0 weeks), the median body weight was 1050 g (IQR: 800-1346 g). Preterm newborns with extremely low body weight (<1000 g) comprised 47% of the group. Detailed clinical characteristics of the study subjects are shown in table I.

**Table I:** Clinical characteristics of the study population. Tabela I. Charakterystyka kliniczna badanej populacji.

	NeoPro study cohort N=936	IVH+ N=462	IVH(-) N=474	P-value IVH+ vs. IVH(-)
Gestational age, week *Wiek płodowy, tygodnie* Mean ± SD Median (IQR)	28 ± 2.4 28 (26.3-30)	27.5 ± 2.4 27.4 (25.6-29.3)	28.7 ± 2.2 29 (27.1-30.3)	< 0.0001
Birth weight, g *Masa urodzeniowa* Mean ± SD *Median (IQR)*	1103 ± 388 1050 (800-1346)	1035 ± 376 970 (750-1260)	1187 ± 383 1170 (900-1470)	< 0.0001
Birth weight <1000 g *Masa urodzeniowa<1000 g* N(%)	461 (47.0)	255 (55.2)	172 (36.7)	< 0.0001
Sex *Płeć* male, N(%) *męska*	540 (55.3)	265 (58.0)	247 (52.6)	0.1102
Place of birth *Miejsce urodzenia* inborn, N(%) *urodzone na miejscu* outborn, N(%) *transportowane*	835 (88.9) 104 (11.1)	397 (89.8) 45 (10.2)	396 (87.6) 56 (12.4)	0.3487
Method of delivery *Sposób rozwiązania ciąży* Cesarean section, N(%) *Cięcie cesarskie* Vaginal delivery, N(%) PSN	763 (78.0) 215 (22.0)	336 (73.4) 122 (26.6)	399 (84.7) 72 (15.3)	< 0.0001
5 min. Apgar score *Ocena wg. Apgar w 5 min*. Mean ± SD *Median (IQR)*	6.4 ± 2.1 7 (6-8)	5.9 ± 2.2 6 (5-7)	6.9 ± 1.8 7 (6-8)	< 0.0001
Antenatal steroids *Steroidy prenatalne* N(%)	762 (78.4)	356 (78.0)	368 (79.0)	0.7525

In the IVH+ group there were no data available for the following number of newborns: sex (n=5), place of birth (n=20), method of delivery (n=4), Apgar score (n=18), antenatal steroid therapy (n=5); in the IVH(-) group: birth weight (n=5), sex (n=4), place of birth (n=22), method of delivery (n=3), Apgar score (n=16), antenatal steroid therapy (n=8).

Data were collected using a paper-based questionnaire in tertiary (n=42) and secondary reference neonatal intensive care units (n=5), between November 2014 and December 2015.

Inclusion criteria were: (1) gestational age ≤ 32 weeks; (2) diagnosis of RDS regardless of the degree of radiological findings in the lungs; and, (3) need for exogenous surfactant. The exclusion criterion was the presence of a clinically significant congenital defect. The study protocol was approved by the Bioethics Committee of the Medical University of Warsaw.

The following factors were considered as potentially influencing the incidence of IVH: antenatal steroids, method of delivery, time elapsed prior to umbilical cord clamping, umbilical cord milking, time of initiation of caffeine citrate, use of invasive ventilation, and duration of invasive ventilation.

Treatment with caffeine citrate was not explicitly defined in the study protocol and the time of its initiation depended upon the practice at the specific site. The group of patients receiving “early” caffeine treatment was defined as those infants in whom caffeine citrate was initiated within the first 24 hours of life; patients receiving “late” caffeine were those who initiated this treatment on the second day of life or later.

IVH was diagnosed using trans-fontanel ultrasonography performed according to the approved standards and further classified using Papile’s grading system [5, 6].

Two-tailed Student’s t-test (in the case of normal distribution) or U-Mann-Whitney test (non-normal distribution) were used, as appropriate, to compare the means between independent variables. For comparisons of percentages, chi-square test or Fisher’s exact probability test were employed. The significance level (alpha) was set at 0.05, with p-values less than alpha considered to be statistically significant.

## Results

### Intraventricular hemorrhage

**Fig. 1:**
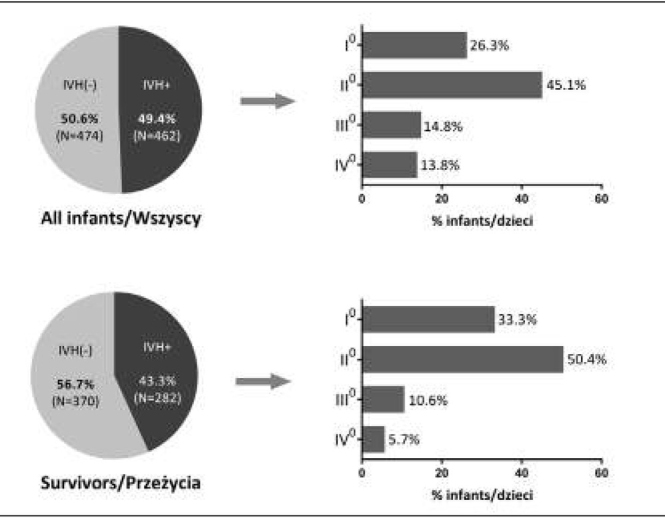
Incidence and classification of intraventricular hemorrhage in the study population (upper chart) and in the subgroup of survivors (lower chart). Ryc. 1. Częstość i nasilenie (stopień) krwawienia wewnątrzczaszkowego w badanej populacji (górny wykres) i w podgrupie dzieci, które przeżyły (dolny wykres).

IVH was diagnosed in 462/936 (49.4%) newborns, while hemorrhages that were severe and had a poor prognosis occurred in approximately 28.6% newborns, including grade 3 hemorrhage in 14.8% and grade 4 in 13.8% infants (fig. 1).

An additional analysis of incidence specifically focused on survivors (652 infants).The incidence rate of hemorrhage in this subpopulation was 43.3% (282 newborns). In this group, hemorrhages that were severe and had poor prognosis were diagnosed in 10.6% newborns (grade 3), and 5.7% newborns (grade 4) (fig. 1).

Out of 139 newborns who died during the observation period the information concerning central nervous system hemorrhage was not available for 19 infants. As for the remaining 120 deaths, IVH was diagnosed in 69.2% of the cases. It is not known whether IVH was the cause of death, as this information was not captured in the study records. It is of note, however, grade 3 or 4 IVHs were predominant in this subgroup and accounted for 73.5% of all IVHs cases. As for the children excluded from the study because they were transferred to another ward or hospital, hemorrhage was diagnosed in 61.4% of the cases.

### Factors potentially influencing the risk of IVH

#### I Antenatal corticosteroids

A full course of antenatal steroids was administered to 762 mothers (78.4%). after analyzing the effect of steroid therapy on the overall incidence rate of intraventricular hemorrhage, no statistically significant association was observed. In the group of newborns exposed and not exposed to antenatal steroids during the prenatal period hemorrhage occurred in 46.7% and 48.1% cases, respectively (p=0.7489). We noted a statistically significant positive association with the incidence of most severe hemorrhage here. Antenatal steroid therapy significantly reduced the incidence of grade 3 and 4 hemorrhage from 22.1% in newborns not exposed to steroids to 14.2% in newborns exposed to steroids (OR: 0.58, 95% CI: 0.39-0.87).

**Fig. 2:**
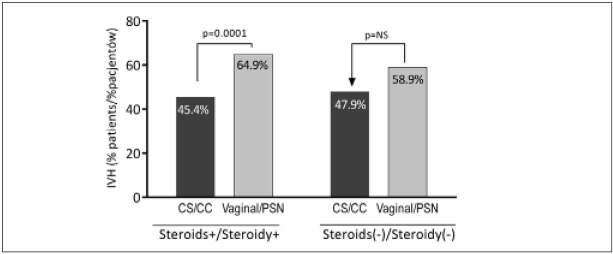
Incidence rate of IVH in infants born (vaginal delivery, or cesarean section), by exposure to antenatal cortycosteroid therapy. Ryc. 2. Częstość występowania IVH u dzieci urodzonych drogami natury i przez cesarskie cięcie, w zależności od zastosowania kortykoterapii prenatalnej. CC = cesarean section

In newborns who were administered antenatal corticosteroids, the method of delivery had a significant impact on the presence of hemorrhage. In infants born naturally, IVH was much more common than in newborns born by cesarean section (64.9% vs. 45.4%; p=0.0001). Such dependency was not observed in newborns who did not receive antenatal steroid therapy (p>0.05; fig. 2).

#### II Method of delivery

In the group that was analyzed, the incidence rate of intraventricular hemorrhage was 45.7% for cesarean section, and 62.9% for natural births.The difference was statistically significant (p<0.0001).

#### III Time to umbilical cord clamping

The time that elapsed before umbilical cord clamping and cord milking did not significantly affect the incidence rate of IVH. In the group where the umbilical cord was clamped earlier than 60 seconds after birth, the incidence rate of hemorrhage was 49.6%. If the cord was clamped after 60 seconds, the incidence rate was 43.9% (p=0.288).

In newborns who underwent ‘cord milking’, the incidence rate of hemorrhage into the central nervous system was 51.1%. If cord milking was not performed, the incidence rate was not significantly different (48.2%; p=0.4192).

#### IV Starting time of treatment with caffeine

Intraventricular hemorrhage occurred significantly more often (57.6%) in the group of newborns where caffeine treatment was started on the second day of life or later. In the group treated “early”, the incidence rate was 46.1% (p=0.0094). Early administration of caffeine significantly reduced the risk of hemorrhage (OR: 0.63, 95% CI: 0.450.88). However, it did not significantly reduce the incidence of severe intraventricular hemorrhage compared to “late” treatment (13.8% and 11.4%, respectively; p=0.4480).

#### V Duration of mechanical ventilation

In the group of newborns receiving mechanical ventilation, the incidence rate of IVH was significantly higher (54.8%) than in newborns who were not mechanically ventilated (29.4%; p<0.0000). Also, if the mechanical ventilation lasted longer than seven days, the incidence of hemorrhage was significantly higher, both in comparison to newborns mechanically ventilated for less than seven days (70.3% vs. 44%; p<0.0000) and those who were not mechanically ventilated at all (70.3% vs. 29.7%; p<0.0000).

**Fig. 3:**
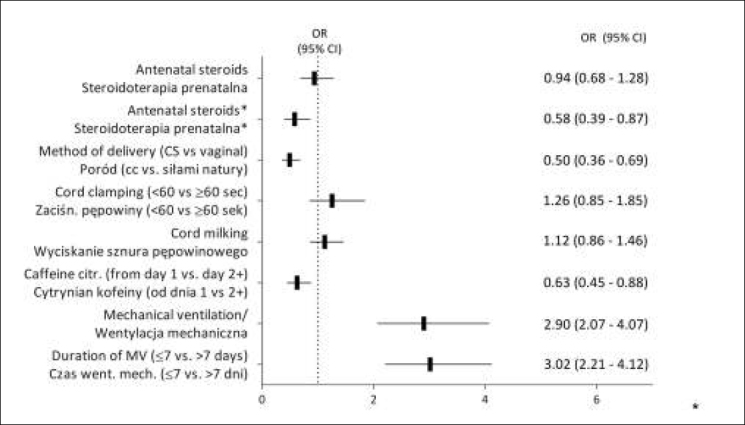
The impact of the factors analyzed on the risk of intraventricular hemorrhage. Ryc. 3. Wpływ analizowanych czynników na ryzyko krwawień wewnątrzczaszkowych.

The impact of potential factors in&uencing the risk of IVH with corresponding odds ratios is shown in figure 3.

* *refers to grade 3 and 4 hemorrhage

## Discussion

The aim of this study was to estimate the incidence rate of intraventricular hemorrhage in newborns with respiratory distress syndrome treated with surfactant, and to identify the factors that might be in&uential in lowering the risk of intracranial hemorrhage in a prospective study of infants born before 32 gestational weeks.

Despite the fact that treatment of extremely preterm infants has evolved in the last 20 years, the problem of IVH is still present. Based on the data obtained from EU countries, the incidence rate of severe hemorrhage (measured as the sum of grade 3 IVH and PVHI) ranges from 2% do 25% in the population at risk consisting of preterm infants born before 32 weeks’ gestation (7).

The main protective factor during the fetal period, which reduces the incidence of intraventricular hemorrhage, is the antenatal administration of steroids.The administration of a full course of corticosteroids reduces the incidence rate of postnatal hemorrhage into the brain ventricles by two to three times, compared to infants whose mothers did not receive this treatment, or mothers who were not administered the full course [8, 9, 10, 11, 12]. Corticosteroids are beneficial, as they facilitate lung development, which makes it possible to achieve cardiorespiratory stabilization after birth, and also helps the maturation of cerebral blood vessels, in which damage could potentially lead to IVH.The refers mainly to an extensive vascular network that nourishes the stem substance located underneath the lateral ventricles. Our study showed that in the absence of antenatal corticosteroids there was a statistically significant increase in the incidence rate of severe hemorrhage from 14.2% to 22.1%. This emphasizes the importance of this treatment and should encourage promoting the treatment among obstetric and neonatal professionals, according to the established standards.

Data concerning the risk of IVH based on the method of delivery is contradictory, and many studies reporting such data were written during a period when the use of antenatal corticosteroids was still limited. Prolonged labor and breech births may lead to negative hemodynamic effects, while a cesarean section performed before the start of uterine contractions seems to act as a protective factor [13, 14]. However, studies concerning the development of infants born with extremely low birth weight (ELBW) indicate that the labor method is not a significant factor that generates IVH [15]. Obstetricians’ experience shows that in case of extreme prematurity (23-25 gestational weeks) performing a cesarean section on a uterus that is not yet prepared for labor may cause the uterus to constrict on the fetus, thus making it hard to extract the baby, which, in turn, raises the risk of intracranial hemorrhage in the newborn.

Recent studies show that additional transfusion of blood from the placenta to the infant born prematurely using delayed umbilical cord clamping, or by removing blood from the umbilical cord reduces the risk of IVH (41% fewer cases of IVH). This is beneficial from the hemodynamic point of view and enables better filling of the placenta with blood [16, 17]. In our study, we failed to confirm this effect. However, the lack of observable effect may result from the fact that in the study patients the delayed umbilical cord clamping was performed only in a small group of newborns (14.9%).

caffeine is one of the basic medications used in neonatology and is considered the ‘gold standard’ in the prevention and treatment of apnea of prematurity. It has been noted that caffeine reduces the incidence rate of bronchopulmonary dysplasia (BPD), patent ductus arteriosus, and also contributes to better development of these newborns in future [18]. Recently, a study by Taha et al. proved that when the medication is administered ‘early’ (no later than in the third day of life), the incidence rate of severe IVH is significantly lower [19].

In our study caffeine administration during the first 24 hours of life very often meant that the medication was given during the first few hours after birth and even directly after birth, which significantly reduced the risk of IVH.The indicates the need to continue the population-based studies in this area. Most intracranial hemorrhage (more than 90%) occur in the first week after birth, thus mechanical ventilation of the infant that takes longer than seven days should not raise the risk of IVH. Higher incidence rates of intraventricular hemorrhage in infants who were ventilated for a longer time period may indicate that in newborns whose condition was more severe and harder to quickly stabilize, IVH occurs more often, and the condition of their lungs does not allow to cease the ventilation earlier.

## Conclusions

The results of our study confirmed that the pharmacological treatment of a fetus with corticosteroids administered to mothers at risk of preterm labor has a protective effect on the risk of severe manifestations of IVH. In addition, the method of delivery was important in newborns exposed to corticosteroids; natural birth was associated with an elevated risk of IVH. Mechanical ventilation was a major risk factor. Finally, early (from the first 24 hours of life) caffeine treatment reduced the risk of bleeding into the central nervous system.

## References

[1] Volpe J (2008). Intracranial hemorrhage: germinal matrix-intraventricular hemorrhage, in: Neurology of the newborn, 5rd edition.

[2] Ballabh P (2010). Intraventricular hemorrhage in premature infants: mechanism of disease. Pediater Res.

[3] Perlman J, McMenamin J, Volpe J (1983). Fluctuating cerebral blood flow velocity in respiratory-distress syndrome. Relation to the development of intraventricular haemorrhage. N Engl J Med.

[4] Sameer Y, Maryam A (2014). A systematic review and meta-analysis of the timing of early intraventricular hemorrhage in preterm neonates: clinical and research implications. J Clin Neonat.

[5] Helwich E, Bekesińska-Figatowska M, Bokiniec R (2017). Standard badań obrazowych ośrodkowego układu nerwowego noworodka, w: Standardy opieki medycznej nad noworodkiem w Polsce. Zalecenia Polskiego Towarzystwa Neonatologicznego, wyd. II.

[6] Papile L, Burstein J, Burstein R, Koffer H (1978). Incidence and evolution of subependymal and intraventricular hemorrhage: a study of infants with birth weights less then1500 gm. J Pediatr.

[7] (2008). Euro-Peristat Project, Perinatal Health Report.

[8] Roberts D, Dalziel S (2006). Antenatal corticosteroids for accelerating fetal lung maturation for women at risk of preterm birth (review). Cochrane Syst Rev.

[9] Wong D, Abdel-Latif M, Kent A; NICUS Network. (2014). Antenatal steroid exposure and outcomes of very premature infants: a regional cohort study. Arch Dis Child Fetal Neonatal Ed.

[10] Ment L, Oh W, Ehrenkrantz R (1995). Antenatal steroids, delivery mode and intraventricular hemorrhage in preterm infants. Am J Obst Gynecol.

[11] Leviton A, Kuban K, Pagano M (1993). Antenatal cortycosteroids appear to reduce the risk of postnatal germinal matrix hemorrhage in intubated low birthweight newborn. Pediatrics.

[12] Almeida B, Rios L, Araujo E (2017). Antenatal corticosteroid treatment for the prevention of peri-intraventricular haemorrhage in preterm newborns: a retrospective cohort study using transfontanelle ultrasonography. J Ultrason.

[13] Wadhawan R, Vohr B, Fanaroff A (2003). Das labor in&uence neonatal and neurodevelopmental outcomes of ELBW infants who are born by cesarean delivery?. Am J Obstet Gynecol.

[14] Gawade P, Whitcomb B, Chasan-Taber L (2013). Second stage of labor and intraventricular hemorrhage in early preterm infants in the vertex presentation. J Matern Fetal Neonatal Med.

[15] Bhatta S, Keriakos R (2011). Review of the recent literature on the mode of delivery for singleton vertex preterm babies. J Pregnancy.

[16] Mercer J, Vohr B, Ericson-Owens D (2010). Seven-month developmental outcomes of very low birth weight infants enrolled in randomized controlled trial of delayed versus immediate cord clamping. J Perinatol.

[17] Christensen R, Carroll P, Josephson C (2014). Evidence-based advances in transfusion practice in neonatal intensive care units. Neonatology.

[18] Schmidt B, Anderson PJ, Doyle LW (2012). Survival without disability to age 5 years after neonatal caffeine therapy for apnea of prematurity. JAMA.

[19] Taha D, Kirkby S, Nawab U, Dysart KC, Genen L, Greenspan JS, Aghai ZH (2014). Early caffeine therapy for prevention of bronchopulmonary dysplasia in preterm infants. J Matern Fetal Neonatal Med.

